# Structural Engineering of Core–Shell Ni_3_B@Ni(BO_2_)_2_ on V_2_MoO_8_ (0D@2D/1D) Composites: Advanced Strategies for Enhancing High Energy Density in Asymmetric Supercapacitors

**DOI:** 10.1021/acs.langmuir.5c00378

**Published:** 2025-04-21

**Authors:** Ahamed Milton, Abdullah Al Mahmud, Ramaraj Sukanya, Raj Karthik, Eswaran Kamaraj, Carmel B. Breslin, P. Muhammed Shafi, Jae-Jin Shim

**Affiliations:** † School of Chemical Engineering, Yeungnam University, Gyeongsan, Gyeongbuk 38541, The Republic of Korea; ‡ Department of Chemistry, Maynooth University, Maynooth, County Kildare W23 F2H6, Ireland; § Department of Chemistry, Yeungnam University, Gyeongsan, Gyeongbuk 38541, The Republic of Korea; ∥ Department of Physics, National Institute of Technology Calicut, Calicut 673601, Kerala, India

## Abstract

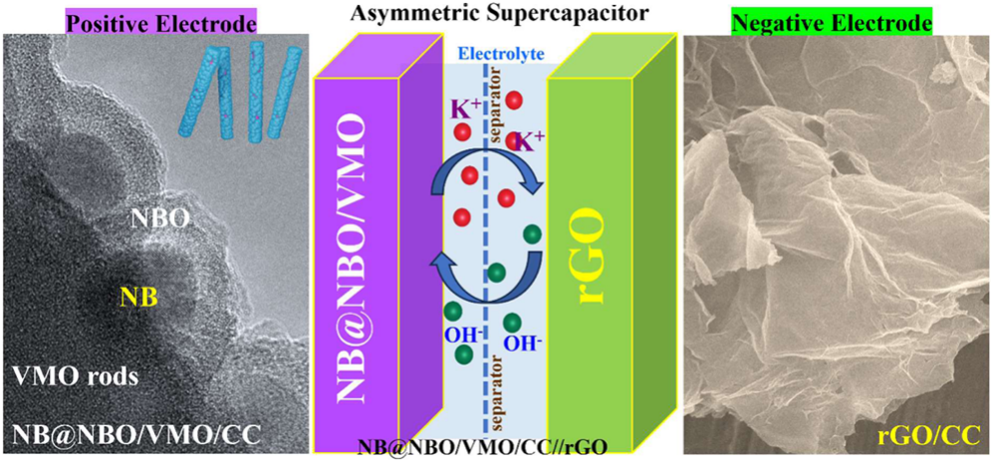

The development of hierarchical core–shell structures and multicomponent metal boride/metal oxide-based composites presents a promising strategy to enhance supercapacitor (SC) performance. In this study, we synthesized a Ni_3_B@Ni(BO_2_)_2_ (0D@2D) core–shell structure and integrated it with V_2_MoO_8_ (VMO) rods (1D) to form a Ni_3_B@Ni(BO_2_)_2_/VMO (NB@NBO/VMO (0D@2D/1D)) composite. This composite was then used as an electrode material on a flexible carbon cloth (CC) substrate for SC applications. The 1D-VMO rods were derived from V-doped MoSe_2_ nanosheets via hydrothermal synthesis and calcination, while the NB@NBO/VMO composite was obtained by using a liquid-phase method. Structural, compositional, and morphological characterizations were conducted using XRD, XPS, FE-SEM, and TEM-EDS. In a three-electrode system, the NB@NBO/VMO-50 composite showed an impressive *C*_s_ of 698 F g^–1^ at 1 A g^–1^, ascribed to its unique core–shell architecture, which enhances contact and faradaic properties, shortens ion diffusion paths, and provides abundant active sites. Notably, the NB@NBO/VMO-50 displayed excellent cyclic stability, retaining 75.1% of its capacitance after 10,000 cycles at 10 A g^–1^. This performance is better than those of other electrodes, including pristine VMO/CC, NB/CC, NB@NBO/VMO-25, and NB@NBO/VMO-75. When evaluated in a two-electrode asymmetric SC system, the NB@NBO/VMO-50/CC||rGO device operated at 1.6 V and delivered a high energy density (ED) of 40.5 Wh kg^–1^ at a power density (PD) of 800 W kg^–1^. It also reached a PD of 16,000 W kg^–1^ while maintaining an ED of 23.5 Wh kg^–1^. The device also showed remarkable long-term durability, maintaining 79.3% of its capacitance and 99.9% Coulombic efficiency after 8000 charge–discharge cycles at 8 A g^–1^, demonstrating its strong potential for next-generation energy storage applications.

## Introduction

As the world moves towards green and sustainable energy to achieve net-zero emissions, developing clean and renewable sources such as solar, wind, geothermal, and tidal energy has become essential. However, these sources are often seasonal and provide unstable outputs, which limit their direct use due to the constant demand for uninterrupted energy supply in daily life and industrial activities. Consequently, effective energy storage systems are crucial for achieving optimal storage capacity and ensuring reliable electricity supply.^1^ Electrochemical energy storage technologies, including batteries and supercapacitors (SCs), offer promising solutions for powering portable electronics.^2^ SCs have attracted widespread attention for their ability to provide high energy and power densities along with outstanding cycling stability.^3−5^ However, the energy density (ED) of SCs remains moderately lower than those of modern battery systems. Therefore, developing highly effective electrode materials is critical for enhancing the energy density of SCs, and this remains a key focus for both academic research and industrial applications.^6^ In terms of suitable electrode materials, transition metal compounds have been developed as promising active electrode materials, owing to their rich redox activity, excellent structural stability, and fast charge-transfer capabilities. These compounds are extensively studied in various forms, including hydroxides,^7^ phosphides,^8^ sulfides,^9^ and transition metal oxides.^10^ Recently, transition metalloids such as borides (B) have attracted growing attention, driven by the promising redox behavior previously demonstrated by phosphides and nitrides in SCs. These materials are expected to have higher intrinsic conductivity, larger theoretical capacitance, and pronounced redox electroactivity compared to oxides.^11,12^ Transition metal borides (TMBs) exhibit a shorter ion diffusion path length, which leads to the better utilization of the active sites.^13^ This property makes them promising electrode materials for high-performance SCs.

In addition, boron-based materials have attracted substantial attention owing to their environmentally friendly nature, high energy efficiency, and lower cost compared to other nonmetals like sulfur and phosphorus.^14−19^ Similarly, nickel-based materials have also received significant attention as pseudocapacitive electrode candidates, thanks to their strong thermal and chemical stability across different electrolytes, high theoretical capacitance, straightforward synthesis methods, affordability, and environmentally friendly characteristics.^20^ Consequently, Ni_*x*_B is a promising electrode material for SCs. For example, Tripathy et al. synthesized amorphous NiB using a metal–organic framework (MOF) precursor, achieving an impressive specific capacitance of 2580 F/g and a high energy density of 72.55 Wh kg^–1^.^21^ However, metal borides often develop unstable metal borate layers during electrochemical processes, leading to structural failure and decreased conductivity.^22^ To address this challenge and enhance the scalability of Ni_*x*_B electrodes, researchers have explored strategies to mitigate aggregation and structural failures. One effective approach involves designing composite or hybrid structures, with core–shell configurations emerging as a particularly promising strategy. The core–shell architecture consists of a core and a shell composed of different materials, utilizing their combined properties to create a synergistic effect. This structure enhances electrical conductivity and accelerates ion-electron diffusion, facilitating the gradual charge/discharge process. Specifically, the core–shell design provides an open pathway for electron transport, while enabling rapid electrolyte ion diffusion through well-defined channels. Additionally, the enlarged surface area increases the contact between electrolyte ions and active materials, resulting in more efficient electrochemical reactions essential for energy storage applications.^23,24^ Furthermore, encapsulating active electrode materials within a core–shell architecture enhances structural stability by incorporating a protective surface layer. These features make core–shell structures particularly advantageous for electrochemical capacitors, where achieving high specific capacitance, improved capacitive retention, and mechanical stability is essential. For example, Cao et al. designed a tightly bonded nickel boride and graphene oxide hybrid structure to enhance the efficiency of SC applications.^25^ Chen et al. created a core–shell structured amorphous Ni_*x*_B on silicon nanowires and carbon nanowalls as a promising electrode for micro-SCs.^26^ Similarly, core–shell designs with carbon nanotubes^27^ and nanorods^28^ have been explored. One effective approach to address these issues is to grow Ni_*x*_B on transition metal oxides. This method facilitates the creation of composites, significantly enhancing pseudocapacitive performance.^12^ Notably, oxide materials offer superior stability compared to conducting polymers and higher energy density than carbon-based materials.^29^

Bimetallic oxides have garnered significant attention due to the synergistic effects between the two metal elements, which result in increased active sites, enhanced stability, and improved electrical conductivity.^30^ These materials exhibit a wide range of oxidation states and redox processes, making them promising candidates for SC applications.^31^ As a result, binary metal oxides are considered to be key materials for developing future electrochemical energy storage devices. Among transition metals, vanadium and molybdenum are particularly noteworthy due to their rich oxidation states, strong theoretical capacitance, and they conduct electricity well.^32^ For example, Jiang et al. prepared V_0.13_Mo_0.87_O_2.93_ nanowires that exhibited outstanding rate capability, retaining 91.5% of their capacitance at 10 A g^-1^ and maintaining 97.6% of their capacity after 10,000 charge/discharge cycles.^33^ Additionally, Ankinapalli et al. prepared MoV_2_O_8_/MoO_3_ microclusters, which exhibited a remarkable capacitance of 2844 F g^-1^ at 1 A g^-1^, an ED of 37.06 Wh kg^–1^, and a cyclic stability of 128%.^34^ Recent research highlights the exceptional electrochemical performance of vanadium molybdenum oxide (VMO) materials in SCs.^35^ To further enhance this performance and mitigate the aggregation issues associated with Ni_*x*_B in SC applications, strategies involving the development of composites or the decoration of Ni_*x*_B with a VMO have been explored. One effective approach involves integrating metal borides with support materials to prevent their dissolution or structural collapse in electrolytes. This is often achieved by growing metal borides on transition metal oxide substrates. For example, Hou et al. synthesized amorphous CoB nanoflakes on NiMoO_4_ nanorods, creating a NiMoO_4_@amorphous CoB heterostructure that was utilized as a cathode electrode in SCs.^36^ This NiMoO_4_@Co-B delivered a consistent capacity of 236.2 mA h g^–1^ at 0.5 A g^-1^, along with an excellent rate performance of 171.2 mA h g^–1^ at 20 A g^-1^. Karthik et al. reported an amorphous Ni_*x*_B||MnMoO_4_ heterostructure as an electrode for asymmetric SCs. The heterostructure delivered a *C*_s_ of 104 F g^-1^ at 1 A g^-1^ and achieved an impressive ED of 32.5 Wh kg^–1^ along with a power density of 750 W kg^–1^.^37^

Given the challenges associated with synthesizing VMO and drawing from both the existing literature and our own findings regarding the performance of nickel boride in SCs, we aimed to prepare a core–shell nickel boride@metaborate (NB@NBO) structure decorated on VMO rods using a simple synthesis approach for SC applications. This composite material, featuring a multidimensional architecture (0D@2D/1D), demonstrates significantly enhanced electrochemical performance, including improved specific capacitance, energy density, and cycling stability. These enhancements result from the combined effects of different components, each contributing in a unique way: (i) the one-dimensional (1D) VMO structure offers an extensive surface area with easy access for electrolyte ions, which promotes efficient charge transfer and improves the overall performance of electrochemical energy storage; (ii) the core–shell architecture, with NB encapsulated in the protective NBO shell, offers structural stability. This design prevents the degradation of the active material (NB), ensuring long-term cycling stability in SC applications. The multidimensional (0D@2D/1D) configuration not only improves the electrochemical performance but also introduces a highly stable and efficient material design for energy storage applications. The integration of distinct material properties, such as the high surface area of the VMO rods and the protective shell of the NBO, leads to synergistic effects that optimize the charge storage and cycling stability of the SC.

In this work, a simple and economical approach was used to synthesize NB@NBO/VMO composites, which served as positive electrode materials for asymmetric SC applications. Notably, the VMO rods serve as a substrate, anchoring the core–shell NB@NBO structure and preventing its agglomeration during the synthesis. The synthesized NB@NBO/VMO composites were thoroughly characterized by using various techniques. The composite materials, featuring a multidimensional architecture (0D@2D/1D), demonstrated enhanced electrochemical performance, including improved specific capacitance and energy density along with reduced resistance and enhanced cycling stability. Optimal performance was achieved by varying the VMO content (i.e., 25, 50, and 75 mg) in the NB@NBO/VMO composites within a three-electrode system. Using the optimized composition, we constructed an ASC device with reduced graphene oxide (rGO) as the anode and NB@NBO/VMO as the cathode on carbon cloth (CC). The high electrochemical performance of the NB@NBO/VMO/CC||rGO SCs device is attributed to the synergistic effects within the composite, resulting in impressive energy density and rate capability. The unique properties of the as-prepared composites and their application in SCs are likely to stimulate an increased research interest in future energy storage technologies.

## Experimental Sections

### Chemicals

Vanadium chloride (VCl_3_, 99%), nickel(II) chloride hexahydrate (NiCl_2_·6H_2_O, 99.3%), 1-methyl-2-pyrrolidone (NMP, 99.0%), acetylene black, and poly(vinylidene fluoride) (PVDF, (−CH_2_CF_2_–)*_n_*) were purchased from Alfa Aesar. Sodium borohydride (NaBH_4_, 98.0%), sodium molybdate dihydrate (Na_2_MoO_4_·2H_2_O, 99.5%), and selenium powder (Se, 99.5%) were obtained from Sigma-Aldrich. Sodium hydroxide (NaOH), ethanol (C_2_H_5_OH, 99.5%), hydrazine monohydrate (N_2_H_4_·*x*H_2_O, ≥97%), and potassium hydroxide (KOH, 85.0%) were purchased from Duksan, Samchun, and Daejung, respectively. All the chemicals used in this study were of analytical grade quality and were utilized in their original form without any further purification.

### Synthesis of VMO Rods from 2D V-Doped MoSe_2_ Nanosheets

The 1D VMO rods were derived from 2D V-MoSe_2_ following our previously reported procedure with slight modifications.^38^ Briefly, V-MoSe_2_ was synthesized by using a simple hydrothermal method. Initially, two separate beakers, each containing 30 mL of deionized (DI) water, were prepared with 157 mg of VCl_3_ and 241 mg of Na_2_MoO_4_. Under continuous stirring, the Na_2_MoO_4_ solution was gradually added dropwise to the dissolved VCl_3_ solution and the resulting mixture was stirred for about 15 min. Simultaneously, 157 mg of powdered Se was dissolved in 10 mL of hydrazine monohydrate solution and gradually added to the VCl_3_ and Na_2_MoO_4_ mixture, resulting in a black/dark brown-colored solution. The mixture was then stirred continuously with a magnetic stirrer for 30 min. The prepared solution was then transferred into a 100 mL Teflon-lined stainless-steel autoclave and treated hydrothermally at 180 °C for 12 h. After the system was allowed to cool naturally to room temperature, the resulting dark-colored precipitate was separated via vacuum filtration and carefully washed several times with C_2_H_5_OH and deionized water. Finally, the collected material was dried overnight in an oven at 60 °C to obtain the V-MoSe_2_ nanosheets.

A small portion of the V-MoSe_2_ nanosheets was placed in a quartz boat and heated in a muffle furnace at 550 °C for 4 h in air to form VMO rods. The temperature was increased and decreased at a constant rate of 2 °C per min. Under these conditions, the V-MoSe_2_ nanosheets underwent oxidation, resulting in the formation of VMO rods.

### Synthesis of Core–Shell Ni_3_B@Ni(BO_2_)_2_ on VMO Rods

Initially, a uniform suspension was prepared by dispersing 50 mg of VMO rods in 25 mL of deionized water, followed by sonication for a few minutes. The suspension was then stirred magnetically, and 742 mg of NiCl_2_·6H_2_O was added. The mixture was maintained at 0 °C in an ice bath under a constant flow of N_2_ gas for 15 min. Next, 10 mL of deionized water containing 40 mg of NaOH and 189 mg of NaBH_4_ was slowly added to the mixture, while stirring continuously for 30 min under a constant flow of N_2_ gas. Afterward, the composite materials were then cleaned using deionized water and CH_3_CH_2_OH via vacuum filtration, followed by drying in an oven at 60 °C for 12 h. The VMO rods with amorphous NB@NBO were subsequently calcined in a quartz boat at 300 °C for 2 h using a heating rate of 5 °C/min under a N_2_ gas flow to produce the NB@NBO/VMO-50 composites.

**Scheme 1:**
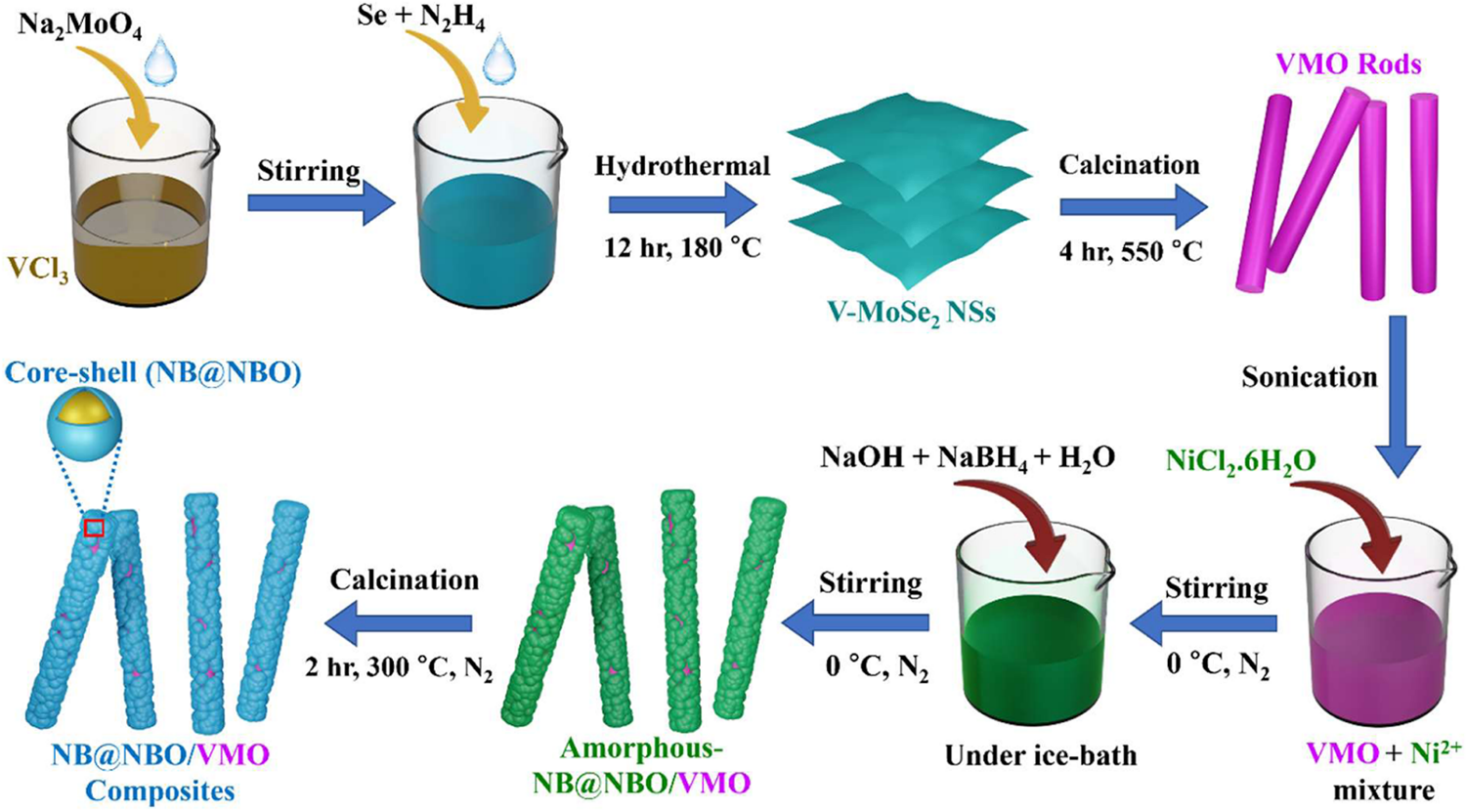
Overall Synthesis Procedure for the VMO Rods and the NB@NBO/VMO Composites

For comparative studies aimed at optimizing the electrochemical performance of the NB@NBO/VMO composites in SCs, the amount of VMO rods was varied to 25 and 75 mg. The resulting composites were designated as NB@NBO/VMO-25 and NB@NBO/VMO-75, respectively. The overall synthesis procedure for the VMO rods and NB@NBO/VMO composites is illustrated in Scheme 1. Additionally, pristine NB was prepared without VMO rods for comparison in SC applications. In this work, rGO was utilized as the anode material for the SC device fabrication. To prepare rGO, we first synthesized graphene oxide (GO) from graphite, followed by the reduction of GO to rGO. Detailed synthesis procedures for these materials are provided in Sections S1.1–S1.3.

### Physicochemical Characterization Techniques

A complete analysis of the chemical, morphological, and structural properties of the as-prepared materials was conducted using various advanced techniques. Detailed characterization methods, including XRD, FE-SEM with EDX, TEM, HR-TEM, and XPS, are provided in Section S1.4.

### Electrochemical Measurements

The electrochemical performance of the synthesized materials was systematically evaluated through CV, GCD, and EIS techniques in both three- and two-electrode configurations. Electrode preparation procedures, testing conditions, and calculation methods for specific capacitance, ED, and PD are described in detail in Section S1.5.^39^

## Results and Discussion

### Structural Characterization of NB, VMO, and NB@NBO/VMO Composites

**Figure 1:**
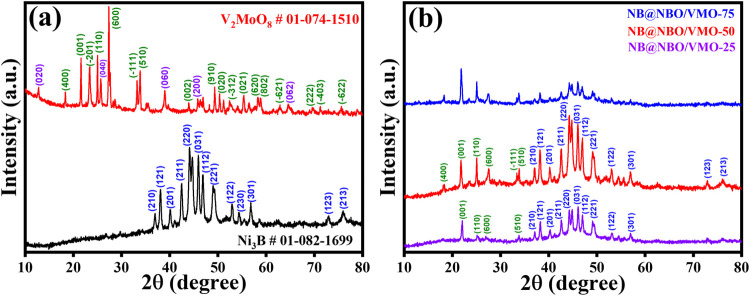
XRD diffractograms of (a) VMO and NB and (b) various ratios of NB@NBO/VMO composites.

The synthesis and confirmation of VMO rods from V-MoSe_2_ nanosheets, achieved through a hydrothermal method followed by calcination, are described in detail in our previous publication.^38^ For comprehensive results and explanations regarding the XRD patterns of the transformation from V-MoSe_2_ to VMO rods, please refer to our earlier work.^38^ The crystalline structures of the prepared VMO, NB, and various core–shell structured composite materials, including NB@NBO/VMO-25, NB@NBO/VMO-50, and NB@NBO/VMO-75, were analyzed using bulk XRD. The XRD patterns for NB and pristine VMO are shown in Figure 1a. For nanocrystalline Ni_3_B, diffraction peaks are observed at 36.9, 38.1, 40.1, 42.5, 44.1, 44.7, 45.9, 46.8, 49.0, 49.4, 53.0, 54.4, 56.7, 58.6, 72.9, and 75.8°, which correspond to the (210), (121), (201), (211), (220), (102), (031), (112), (221), (131), (122), (230), (301), (311), (123), and (213) planes, respectively. These peaks align with the orthorhombic phase of Ni_3_B as referenced by JCPDS card no. 01-082-1699.^40^ Similarly, the XRD pattern for V_2_MoO_8_, shown in the upper part of Figure 1a, includes peaks at 18.3, 21.6, 23.4, 23.5, 24.9, 27.5, 33.2, 33.8, 49.1, 55.3, 58.3, and 58.9°, which correspond to the (400), (001), (−201), (201), (110), (600), (−111), (510), (910), (021), (620), and (802) planes of the C2 space group and the monoclinic crystal phase of V_2_MoO_8_, confirmed by JCPDS card no. 01-074-1510.^41^ Additional peaks with low intensities at 12.9, 25.7, 39.0, and 45.8° are attributed to the MoO_3_ phase, likely due to prolonged calcination during VMO formation. Figure 1b presents the XRD patterns for the different composite ratios, including NB@NBO/VMO-25, NB@NBO/VMO-50, and NB@NBO/VMO-75. It is observed that the intensity of the VMO peaks increases with the amount of VMO (from 25 to 75 mg), while the peaks corresponding to NB diminish. Notably, the MoO_3_ peaks, which were present in the VMO sample, are either significantly diminished or not visible in the composites calcined at 300 °C under a N_2_ atmosphere. The decreased intensity of MoO_3_ peaks can be ascribed to the increased prominence of the VMO peaks and the crystalline or nanocrystalline characteristics of the composite materials. Furthermore, the HR-TEM images in Figure 3c,d confirm the noncrystalline nature of the metaborate, as the lack of a clear XRD pattern in the composites suggests minimal or no crystallinity.

**Figure 2:**
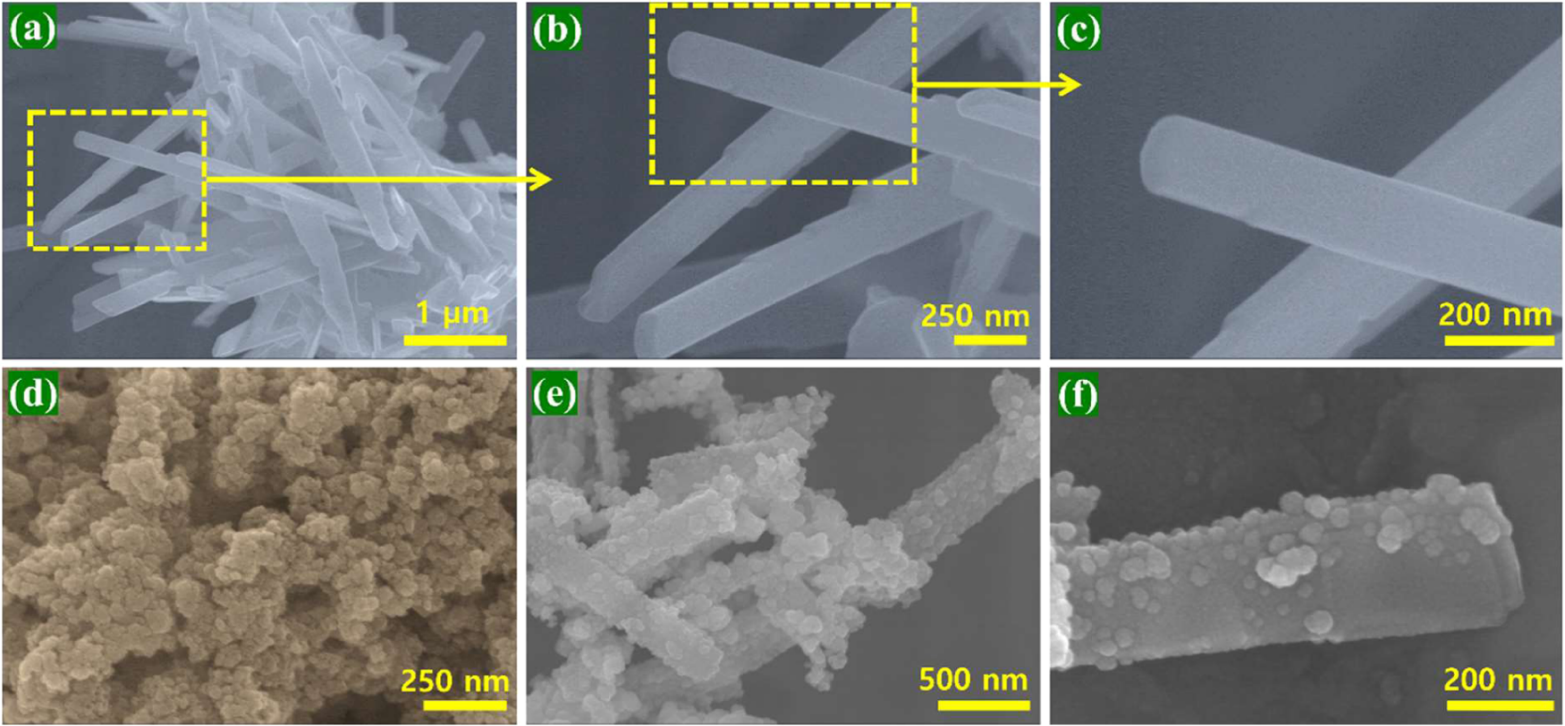
FE-SEM images at various magnifications of (a–c) VMO rods, (d) NB, and (e, f) NB@NBO/VMO-50 composites.

**Figure 3:**
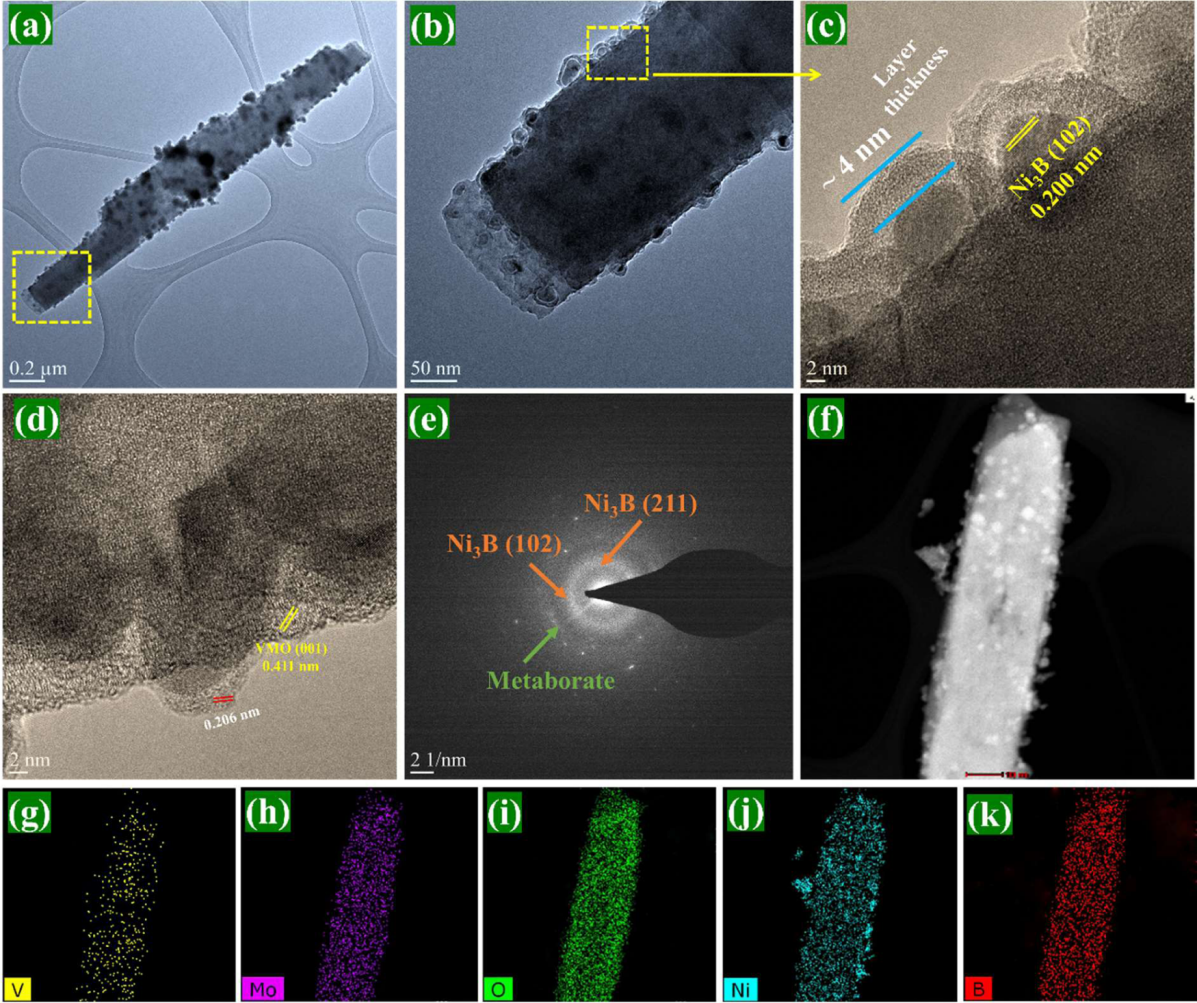
(a, b) TEM images, (c, d) HR-TEM images, (e) SAED pattern, and (f–k) EDX elemental mapping of NB@NBO/VMO composites: (f) HAADF-STEM image, (g) vanadium(V), (h) molybdenum (Mo), (i) oxygen (O), (j) nickel (Ni), and (k) boron (B).

The morphology of the NB, VMO, and NB@NBO/VMO composites was analyzed using FE-SEM and FE-TEM. Figure 2a–c displays both high- and low-magnification images, confirming that VMO exhibits a characteristic rod-like structure with uniform dimensions. Specifically, Figure 2a reveals that the VMO rods have an average length of approximately 2.2 μm and a diameter of about 195 nm. The smooth surface of the rods, as seen in Figure 2c, likely results from the high-temperature calcination process at 550 °C; this contributes to the high crystallinity observed in the XRD analysis. This rod-like structure of VMO, whether in pure form or as part of a composite, provides an optimal surface area for electron transport, enhancing both electrical conductivity and electrochemical kinetics. Figure 2d shows the high-magnification image of pristine NB, which appears as aggregated spherical particles. Figure 2e,f illustrates the successful incorporation of the core–shell NB@NBO structure onto the VMO rods. The surface of the VMO rods becomes less smooth after decoration with NB@NBO, with the rod-like structure being partially covered by the NB@NBO core–shell. Figure 2f further confirms that the VMO rods are adorned with a substantial amount of NB@NBO, significantly increasing the active surface area for electrochemical reactions. The uniform distribution of the NB@NBO core–shell structure on the VMO rods is evident, although the core–shell structure is difficult to distinguish in the SEM images. Therefore, TEM analysis was performed to confirm the core–shell NB@NBO on the rods, with the results presented in Figure 3.

The TEM and HR-TEM images in Figure 3a,b confirm that the VMO rods are effectively decorated with NB particles. The HR-TEM image in Figure 3c reveals an additional layer surrounding the NB particles, forming a core–shell structure. The average thickness of this shell layer is approximately 4 nm, likely corresponding to a metaborate layer. The enlarged image (Figure 3d) displayed lattice fringes with a *d*-spacing of 0.200 nm, which corresponds to the (102) plane of nanocrystalline NB. The *d*-spacing of 0.411 nm represents the (001) plane of the VMO rods, and an additional spacing of 0.206 nm suggests that the metaborate edges gained some nanocrystallinity due to the heat treatment at 300 °C. The formation of Ni_3_B, as the primary product before calcination, and Ni(BO_2_)_2_, as a secondary product from the hydrolysis of BH_4_^–^ (BH_4_^–^ + 2H_2_O → BO_2_^–^ + 4H_2_), suggests that the hydrolysis rate of BH_4_^–^ controlled the thickness of the Ni(BO_2_)_2_ layer. It appears that the formation of NB and NBO was competitive with the reaction kinetics favoring the faster formation of NB. EDX elemental mapping (Figure 3f–k) confirms the presence of all expected elements, including vanadium (V), molybdenum (Mo), oxygen (O), nickel (Ni), and boron (B). Vanadium is less prominent, likely due to the transformation of V-doped MoSe_2_ to VMO rods, where the dopant is present in smaller quantities. The SAED pattern in Figure 3e further validates the amorphous/nanocrystalline nature of the core–shell structure NB@NBO and the crystalline structure of both of them present in the NB@NBO/VMO composites. The SEM and TEM analyses clearly show that the core–shell structure NB@NBO is successfully decorated on the interior and edges of the VMO rods.

**Figure 4:**
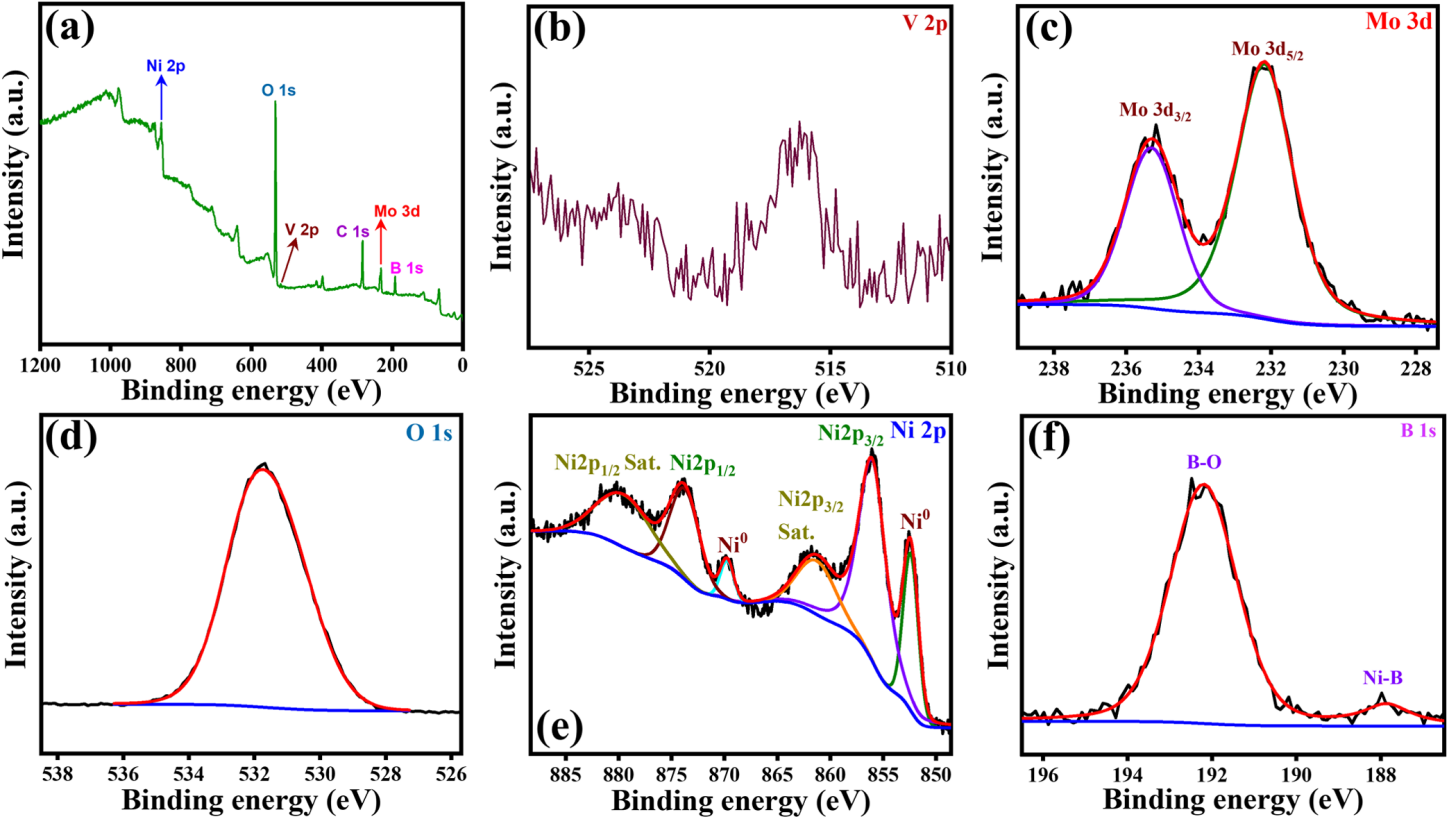
XPS characterization of the NB@NBO/VMO composite, including (a) the full survey spectrum and detailed high-resolution spectra of (b) V 2p, (c) Mo 3d, (d) O 1s, (e) Ni 2p, and (f) B 1s.

Following the morphological and structural analyses, XPS was conducted to investigate the chemical composition and valence states of the synthesized NB@NBO/VMO composites. The XPS overview spectrum (Figure 4a) verifies the existence of Ni, V, Mo, B, and O elements in the composite. Two distinct peaks at binding energies of 873.9 and 856.1 eV are observed in the Ni 2p spectrum (Figure 4e), corresponding to the Ni^2+^ 2p_1/2_ and Ni^2+^ 2p_3/2_ spin orbitals, respectively, indicating the presence of metaborate in the particle shell.^42^ Additionally, the satellite peaks at 880.2 and 861.5 eV are characteristic of Ni^2+^ species, whereas the peaks at 869.7 and 852.5 eV indicate the presence of metallic nickel (Ni^0^) on the surface.^43^ The V 2p spectrum (Figure 4b) reveals primary peaks at 525 and 517.5 eV, corresponding to the V^5+^ state.^44^ A shift to lower energies (523.9 and 516.4 eV) in the composite is observed, indicating the presence of V^4+^ and V^3+^ oxidation states. Peaks observed at 235.3 and 232.2 eV in the Mo 3d spectrum (Figure 4c) correspond to the Mo 3d_3/2_ and Mo 3d_5/2_ core levels, respectively, indicating that molybdenum exists in the Mo^6+^ oxidation state.^41^ In the O 1s spectrum (Figure 4d), the peak observed around 530-532 eV corresponds to lattice oxygen (O^2-^ ions) in V_2_MoO_8_, confirming its incorporation into the composite structure. The B 1s spectrum (Figure 4f) shows peaks at 192.2 and 187.8 eV, associated with B–O and Ni–B interactions in NB.^45^ The observed shift in the binding energy of B from 187.1 eV in pure boron to 187.6 eV indicates electron transfer from boron to nickel, resulting in an increased electron density around the Ni atom. This charge transfer can enhance the electrochemical activity and energy storage. These XPS results confirm the successful synthesis of the NB@NBO/VMO composites.

### Electrochemical Analysis of NB, VMO, and NB@NBO/VMO Composites on CC

**Figure 5:**
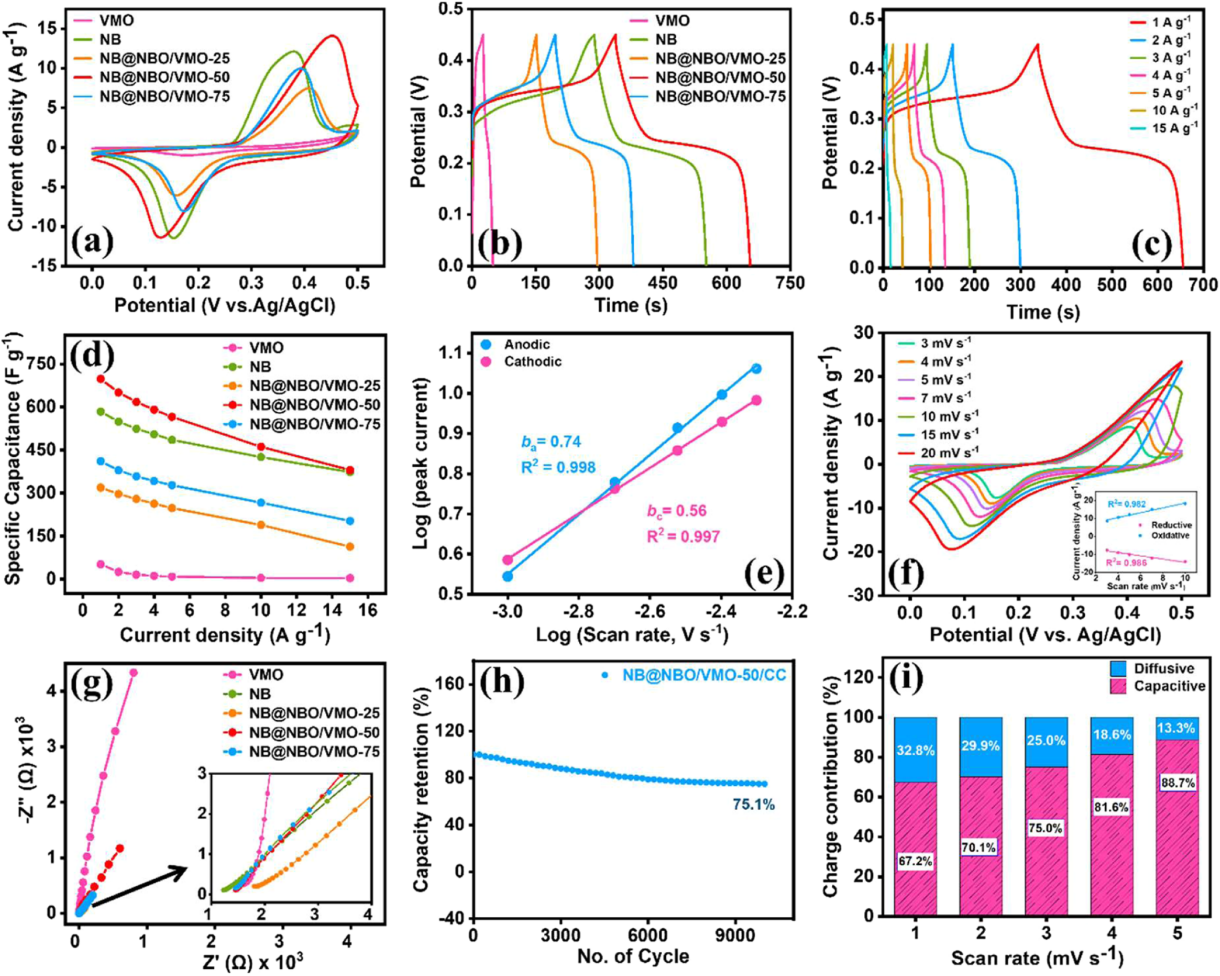
(a, b, d, g) CV, GCD, rate capability plot of the current densities versus *C*_s_, and EIS for different electrodes on the CC substrate. (c) GCD; (e) log–log graph showing the relationship between peak current and scan rate. (f) CV curves at different scan rates (inset: corresponding linear plots for anodic and cathodic peaks); (h) long-term cyclic stability over 10,000 cycles at 10 A g^–1^; and (i) charge separation bar graph for NB@NBO/VMO-50 composites.

Electrochemical investigations were conducted on the prepared NB@NBO/VMO-50 composites with varying compositions, including NB@NBO/VMO-25 and NB@NBO/VMO-75, as well as their individual components (NB and VMO), employing a three-electrode configuration with 3 M KOH as the electrolyte. The analyses included CV at various scan rates (3, 4, 5, 7, 10, 15, 20 mV s^–1^), GCD measurements performed under varying current densities (1–15 A g^–1^), and EIS across a frequency range of 100 kHz to 10 mHz. The CV curves for the composites with different ratios, VMO, and NB electrodes, at a sweep rate of 7 mV s^–1^, as presented in Figure 5a, show that the NB@NBO/VMO-50 electrode exhibits the largest CV loop area, indicating superior electrochemical performance among the prepared electrodes. In contrast, the bare VMO electrode shows the lowest redox peak intensity and smallest CV loop area, suggesting poor faradic behavior. The incorporation of the core–shell structured NB@NBO, known for its conductive and Faradic properties, notably boosts the composite’s electrochemical performance, as evident in Figure 5a. However, the performance of the NB@NBO/VMO-25 and NB@NBO/VMO-75 composites still lags behind that of the bare NB electrode. Notably, the NB@NBO/VMO-50 composite shows a marked improvement, performing better than the bare NB electrode, indicating that this composition is optimal for the composite electrode. At a constant current density of 1 A g^–1^, the GCD profiles of the different electrodes (Figure 5b) display a trend comparable to that seen in the CV studies. All electrodes display almost symmetric charge–discharge curves, suggesting a reversible redox reaction occurring at the electrode–electrolyte boundary. The *C*_s_ of each electrode was calculated from the discharge time using eq S1, with the NB@NBO/VMO-50 composite electrode showing the highest *C*_s_ (698 F g^–1^) among all tested electrodes, including VMO (51 F g^–1^), NB (584 F g^–1^), NB@NBO/VMO-25 (319 F g^–1^), and NB@NBO/VMO-75 (411 F g^–1^), as summarized in Table S1.

The high *C*_s_ of NB@NBO/VMO-50 (698 F g^–1^) is attributed to its well-balanced composition, which integrates the pseudocapacitive properties of VMO with the high conductivity of NB and NBO. The core–shell structure of NB@NBO provides abundant active sites and enhances Faradaic reactions, while VMO introduces additional redox-active centers from vanadium and molybdenum, thereby increasing the electroactive surface area. Furthermore, the porous architecture facilitates efficient ion diffusion and electrolyte penetration, while the conductive NB ensures smooth electron transport. The 50 mg of VMO content achieves an optimal balance between structural and electrochemical stability. In contrast, a lower VMO content (25 mg) results in too few active sites, while a higher content (75 mg) leads to decreased conductivity caused by oxide buildup and NB@NBO aggregation on the VMO surface, thereby hindering access to active sites that are essential for electrochemical reactions. Moreover, the oxidation/reduction endeavor of vanadium and molybdenum, together with the pseudocapacitive contributions of metaborate, further enhances charge storage, making NB@NBO/VMO-50 an excellent electrode material for electrochemical applications. The superior electrochemical energy storage performance of NB@NBO/VMO-50 arises from its multidimensional architecture, which contributes to (i) enhanced specific capacitance and energy density, along with long-term cyclic stability, even in highly alkaline electrolyte conditions; (ii) the synergistic effect of three different materials, each contributing distinct advantages; (iii) the 1D structure of VMO provides a large surface area, enhancing electrolyte ion accessibility and charge transfer kinetics, thereby improving the electrochemical energy storage process; and (iv) the porous core–shell architecture ensures structural stability by encapsulating the active NB material within a protective NBO shell, preventing degradation and maintaining long-term performance. These combined advantages emphasize NB@NBO/VMO-50 as an excellent electrode material for future SCs and high-performance electrochemical energy storage systems.

Further electrochemical assessments, including CV at various sweep rates and GCD at different current densities, were conducted for each electrode, with the detailed results available in the Supporting Information (Figure S1). The bare VMO electrode exhibits a minimal potential gap between the anodic and cathodic redox peaks, indicating a higher reversibility in the charge storage mechanism. Additionally, no significant peak shifts were observed with increasing scan rates, a characteristic feature of electrical double-layer capacitors (EDLCs) and pseudocapacitive materials where surface-limited charge transfer mechanisms dominate.^46^ Conversely, the bare NB and its core–shell composite electrodes (NB@NBO/VMO-25 and NB@NBO/VMO-75) display noticeable potential gaps and peak shifts. Despite this, the NB@NBO/VMO-50 electrode shows a substantial increase in *C*_s_ related to bare VMO and retains over 54.58% (381 F g^–1^ at 15 A g^–1^) of its capacitance even after a 10-fold enhancement in current density. To further assess the enhanced specific capacitance of the NB@NBO/VMO-50 composite, GCD measurements were conducted across a range of current densities from 1 to 15 A g^–1^, as illustrated in Figure 5c. The charge–discharge curves maintain nearly triangular symmetry, indicating excellent reversibility of the electrode. The *C*_s_ values of the NB@NBO/VMO-50 electrode at each current density were calculated as 651 F g^–1^ (2 A g^–1^), 618 F g^–1^ (3 A g^–1^), 591 F g^–1^ (4 A g^–1^), 566 F g^–1^ (5 A g^–1^), 461 F g^–1^ (10 A g^–1^), and 381 F g^–1^ (15 A g^–1^). The slight decrease in *C*_s_ with an increase in current density is likely due to the limited capability of the electrode material to support Faradaic reactions efficiently at higher current rates. Nonetheless, the *C*_s_ of the NB@NBO/VMO-50 electrode remains significantly higher than those of the pristine VMO and NB electrodes, demonstrating its superior performance. This suggests that combining the pseudocapacitive core–shell NB@NBO with the VMO rods enhances charge storage capacity, making it a promising candidate for fast-charging applications. As shown in Figure 5d, the rate capability of NB@NBO/VMO-50 is slightly lower than that of pristine NB. This could be due to the elevated charge transfer resistance resulting from the multicomponent structure, which creates additional interfaces that disrupt electron and ion transport. Moreover, the porous core–shell structure, while enhancing overall capacitance, may limit electrolyte penetration at high current densities. At lower rates, ions fully utilize both surface and bulk redox sites, but at higher rates, restricted ion diffusion leads to a sharper capacitance drop. Additionally, the composite relies more on bulk diffusion-controlled charge storage, whereas pristine NB benefits from faster surface-based redox reactions. Despite this, NB@NBO/VMO-50 continues to deliver enhanced *C*_s_ and long-term stability, demonstrating its potential for use in energy storage applications.

The *C*_s_ versus current density for different electrodes is presented in Figure 5d and Table S2. To further explore the charge intercalation process within the electrode material, a log–log plot was constructed to illustrate the relationship between the peak current and scan rate (Figure 5e). Figure 5f shows the CV profiles of NB@NBO/VMO-50 at various sweep rates, including 3, 4, 5, 7, 10, 15, and 20 mV s^–1^. The corresponding linear plot for the anodic and cathodic peaks is illustrated in the inset of Figure 5f. As the scan rate rises, the anodic peaks tend to shift to higher potentials, while the cathodic peaks move to lower potentials, reflecting a conventional electrochemical behavior associated with rapid voltage sweeps. Interestingly, both anodic and cathodic peak currents follow a general power–law relationship (eq 1), consistent with previous studies.^47^
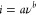
1where the parameters *a* and *b* are adjustable. It is widely recognized that the total measured peak current arises as a result of both diffusion-controlled and surface-controlled charge storage mechanisms. The value of *b* provides insight into the individual contributions to the total current. An *a* value close to 1 (i.e., *i* = *av*) indicates that the capacitive current primarily arises from the surface-limited charge storage mechanism. Conversely, a *b* value close to 0.5 (i.e., *i* = *a*ν^0.5^) suggests that the current is diffusion-limited, indicating that the charge intercalation mechanism dominates.^48^ In this work, the *b* values for both oxidation and reduction processes are below 0.75 (*b*_a_ = 0.74 and *b*_c_ = 0.56, respectively), signifying that the diffusion-limited charge storage mechanism is the predominant contributor to the overall charge storage properties. This enhancement is due to the integration of core–shell NB@NBO, which greatly improves the bulk charge storage capability of the composite electrode, an essential aspect for energy storage device applications.

Moreover, EIS studies further reveal the synergistic behavior of the capacitive VMO and diffusive NB@NBO materials within the NB@NBO/VMO composite electrodes, as illustrated in Figure 5g. The Nyquist plot typically features three distinct regions: at high frequencies, the *x*-intercept signifies the internal resistance (*R*_s_); in the midfrequency range, a semicircle corresponds to the charge transfer resistance; and at low frequencies, the inclined line indicates ion diffusion resistance within the electrode material. Depending on the angle of the vertical slope relative to the *x*-axis, the diffusion resistance can be interpreted as Warburg resistance, a constant phase element, or a capacitive component. The inset of Figure 5g clearly shows that the NB electrode has the lowest internal resistance, indicating its high electrical conductivity. The vertical slope of the bare VMO electrode is nearly 90°, suggesting capacitive behavior, while the slope for the bare NB electrode is close to 45°, indicating semi-infinite diffusive behavior, consistent with previous studies. The NB@NBO/VMO-25-, NB@NBO/VMO-50-, and NB@NBO/VMO-75-modified CC electrodes exhibit an improved internal conductance and moderate diffusion resistance. This provides direct evidence of enhanced electrode conductivity, bulk storage capacity, and smoother charge transfer kinetics due to the incorporation of core–shell NB@NBO onto VMO rods.

Furthermore, the electrodes’ long-term stability was evaluated by subjecting them to 10,000 continuous charge–discharge cycles conducted at a constant current rate of 10 A g^–1^, as illustrated in Figure 5h. As expected, the NB@NBO/VMO-50 electrode maintained a moderate cyclic stability of 75.1% after 10,000 cycles, indicating its capability for long-term use. To further investigate the stability of the NB@NBO/VMO-50 electrode in detail, FE-SEM imaging was conducted after the cyclic stability test, following the methodology outlined in our previous study.^37^Figure S2 presents the FE-SEM image of the electrode following 10,000 charge–discharge cycles carried out at a steady current density of 10 A g^–1^. This image confirms that the electrode has nearly retained its original morphology even after 10,000 charge/discharge cycles in a highly aggressive electrolyte medium. The results indicate that the NB@NBO core–shell structure exhibits a strong binding affinity with the VMO rods, contributing to the enhanced cyclic stability observed after the incorporation of this structure. To further understand the exact contributions of nondiffusion-limited (capacitive) and diffusion-limited charge storage mechanisms to the total charge storage capacity of the NB@NBO/VMO-50 electrode, we quantified the individual contributions using the following relation, eq 2:
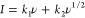
2The total current can be expressed as the sum of contributions from surface-limited capacitive effects, represented by *k*_1_ν, and diffusion-controlled processes, represented by *k*_2_ν^1/2^. The linear plot of *I*/ν^1/2^ versus ν^1/2^ allows for the extraction of coefficients *k*_1_ and *k*_2_, which correspond to the *y*-intercept and slope, respectively. Once these coefficients are known, the individual contributions to the total current can be quantitatively estimated. Figure S3 shows the deconvoluted CV loop areas separating capacitive and diffusive current contributions at different sweep rates (1–5 mV s^–1^). The bar graph in Figure 5i illustrates the capacitive and diffusive contribution percentages of the NB@NBO/VMO-50 electrode. The findings reveal that the composite electrode displays a clear linear trend in the current versus sweep rate plot, suggesting a rapid and efficient charge-transfer process in the NB@NBO/VMO-50 electrode along with substantial bulk charge storage capability. At a scan rate as low as 1 mV s^–1^, the linear component accounts for more than 67% of the total contribution, indicating highly efficient and rapid charge-transfer kinetics. These findings highlight the potential of the NB@NBO/VMO-50 electrode for high-energy, fast-charging storage device applications.

### Device Performance of Core–Shell NB@NBO onto VMO Rods||rGO

**Figure 6:**
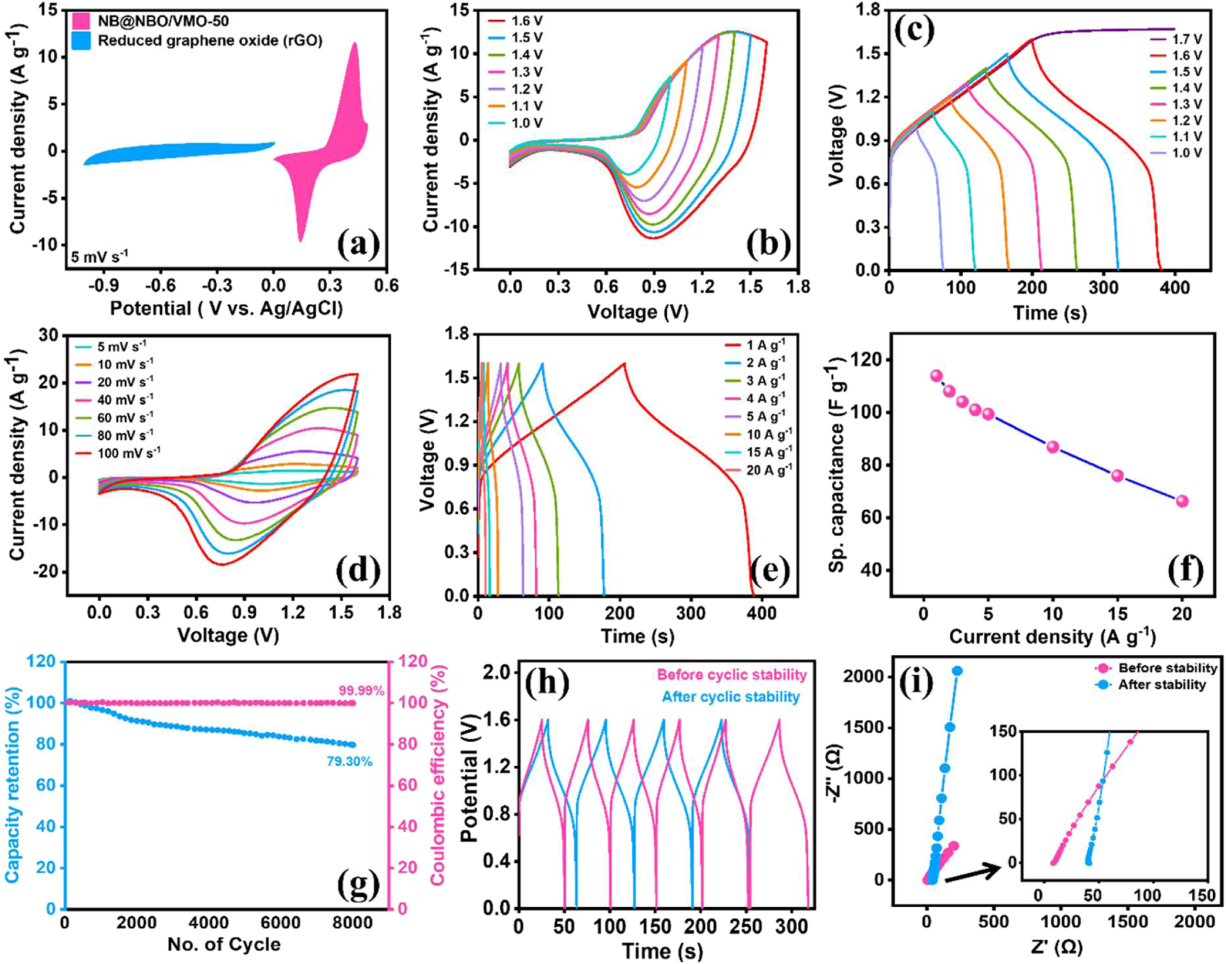
(a) CV of rGO and NB@NBO/VMO-50/CC at 5 mV s^–1^; (b) CV curves at various voltages; (c) GCD profiles at various voltage windows; (d) CV measurements at various sweep rates; (e) GCD measurements at varying current densities; (f) linear plot of *C*_s_ versus current density; (g) cyclic stability over 8000 charge–discharge cycles at 8 A g^–1^; (h) first and last GCD curves for stability studies, and (i) EIS before and after the stability test NB@NBO/VMO-50/CC||rGO (inset: magnified view).

To evaluate the practical potential of the NB@NBO/VMO-50 electrode for energy storage applications, we constructed an ASC device. The ASC device was fabricated by using NB@NBO/VMO-50 as the cathode and rGO as the anode. The mass loading for each electrode was calculated according to eq S2. To establish the ideal operating voltage range for the device, initial CV examinations were conducted at a sweep rate of 5 mV s^–1^ on both electrodes within their individual potential windows, as depicted in Figure 6a. To reveal the feasible operating voltage range of the ASC device, CV measurements were conducted at a sweep rate of 50 mV/s, exploring a potential window from 1.0 to 1.6 V, as shown in Figure 6b. The results show that the CV loop area increases with the expansion of the potential window, indicating an increase in the energy density as the potential range is extended. However, the maximum upper limit of the potential window must be set below the electrolyte decomposition potential where irreversible reactions begin. To establish this limit, GCD profiles were recorded at a constant current density of 1 A g^–1^ with varying upper potential limits, as illustrated in Figure 6c. The GCD curves recorded up to a maximum voltage of 1.6 V exhibit nearly symmetrical charge–discharge profiles, suggesting that the redox reactions occurring during cycling are highly reversible. Consequently, the operating potential window of the NB@NBO/VMO-50/CC||rGO ASC device was set between 0 and 1.6 V. To further investigate the rate capability and fast-charging characteristics of the ASC device, CV measurements were conducted at sweep rates ranging from 5 to 100 mV s^–1^ while maintaining a fixed voltage window of 1.6 V, as presented in Figure 6d. The CV loop area increased with the sweep rate, accompanied by a reasonable shift in the redox peaks, suggesting very good reversibility and an appreciable rate capability. The fast-charging capability of the ASC was evaluated by performing GCD measurements at higher current densities varying from 1 to 20 A g^–1^, as illustrated in Figure 6e.

The GCD profiles demonstrate good symmetry between the charging and discharging phases across all current densities, indicating reversible and rapid charge-transfer kinetics. The ASC device’s *C*_s_ at varying current densities was evaluated based on the formula provided in eq S2, with the only modification being that the mass (*m*) refers to the combined mass of active materials in both the negative and positive electrodes. The resulting capacitance values across different current densities are summarized in Table S3 and illustrated in Figure 6f. At a current density of 1 A g^–1^, the device achieved a high *C*_s_ value of 114 F g^–1^. When tested at 10, 15, and 20 A g^–1^, the capacitance values were 87, 76, and 66 F g^–1^, respectively, corresponding to retention rates of 76.3, 66.6, and 58.4%. These results emphasize the device’s strong performance even under high current conditions.

Moreover, the Coulombic efficiency of 99.99% (almost near 100%) is achieved over 8000 charge–discharge cycles at a current density of 8 A g^–1^, indicating the outstanding reversibility nature of the ASC device. Besides, the cyclic stability over 8000 cycles exhibits a capacitance retention of 79.30% (Figure 6g), indicating the better affinity between core–shell NB@NBO and VMO rods along with a conductive substrate, which improves the wettability of the whole device system. To further evaluate the overall stability of the ASC device, a comparison was made between the charge–discharge profiles from the initial 5 cycles and those obtained after prolonged cycling (cycles 7995–8000), as shown in Figure 6h. While some hysteresis is observed particularly between the early and final cycles, the GCD curves remain nearly symmetrical, with comparable charge and discharge durations, demonstrating stable performance. To validate these observations, EIS measurements were conducted before and after the long-term cycling test. The corresponding Nyquist plots, presented in Figure 6i, reveal a slight increase in internal resistance and a marginal decline in charge transfer efficiency after 8000 cycles, confirming a minor loss in electrochemical performance over time. Furthermore, a more pronounced variation in diffusion is detected at lower frequencies, indicating a slight disruption in the transfer of electrolytic ions within the electrode–electrolyte interface. The EIS analysis (inset of Figure 6i) also shows an increase in bulk resistance (*R*_b_) and charge transfer resistance (*R*_ct_) after the stability test, as seen by the shift of the *x*-intercept to the right and the larger semicircle in the Nyquist plot. The rise in *R*_b_ suggests higher electrode/electrolyte interface resistance, likely due to the formation of passivation layers or electrolyte decomposition. Similarly, the increase in *R*_ct_ indicates a slight decline in charge transfer kinetics, possibly due to surface modifications of the material over prolonged cycling. Despite these changes, the electrode maintains a stable electrochemical response, demonstrating a good structural integrity. These results confirm that while resistance increases after cycling, the core–shell NB@NBO/VMO composite retains its effective charge storage capability, ensuring long-term operational stability. Furthermore, the performance of the ASC device was assessed using a Ragone plot (Figure S4 and Table S4), which illustrates the relationship between ED and PD. Initially, the ED and PD were finalized using eqs S3 and S4. The ED increases as the PD decreases, which aligns with the expected behavior of SCs. These results clearly demonstrate the device’s capability to operate at ultrahigh charging rates, making it a promising candidate for ultrafast charging ASC applications. Additionally, the obtained device performance was compared with existing research studies, as summarized in Table S5. The comparison shows that the current results have better ED and PD, making them comparable to those of advanced SCs. This highlights the enhanced electrochemical properties and practical applicability of the developed ASC device in high-performance energy storage systems.

## Conclusions

In conclusion, we have effectively developed a distinct multidimensional (0D@2D/1D) core–shell NB@NBO structure on VMO rods using a multistep synthesis process and utilized this composite as a positive electrode material for SCs. The electrochemical properties of the NB@NBO/VMO composite were thoroughly examined by adjusting the VMO content within the range of 25–75 mg. Under the optimized conditions, the NB@NBO/VMO-50/CC composite demonstrated exceptional electrochemical performance for SC applications. This composite exhibited significantly enhanced properties compared to pristine NB and VMO rods owing to the synergistic interaction between the core–shell NB@NBO and VMO rods, which contributed to its structural stability and superior performance. The *C*_s_ of NB@NBO/VMO/CC reached 698 F g^–1^ at 1 A g^–1^, demonstrating superior performance compared to the individual NB (584 F g^–1^) and VMO (51.5 F g^–1^) electrodes. To explore practical applications, the high performance of both positive and negative electrodes in the assembled NB@NBO/VMO-50/CC||rGO ASC device resulted in a maximum ED of 40.5 Wh kg^–1^ at a PD of 800 W kg^–1^. These results highlight the commercial and economic promise of the NB@NBO/VMO||rGO ASC device, indicating its viability for advanced energy storage applications.

## Supporting Information Available

Additional experimental data, including chemicals; GO synthesis; RGO synthesis; capacitance comparison of all materials; *C*_s_ of all materials at various current densities; CV and GCD curves of VMO, NB, NB@NBO/VMO-25, and NB@NBO/VMO-75; FE-SEM images of NB@NBO/VMO-50 electrode before and after cyclic stability; CV curves at various sweep rates; capacitance of NB@NBO/VMO-50/CC||rGO at different current densities; ED and PD for the NB@NBO/VMO-50/CC||rGO device; Ragone plot for asymmetric NB@NBO/VMO-50/CC||rGO SC cells; comparison of the performance of the NB@NBO/VMO//rGO with recently reported SCs; references. (PDF)
