# Supplementary material for: Structural Engineering of Core–Shell Ni3B@Ni(BO2)2 on V2MoO8 (0D@2D/1D) Composites: Advanced Strategies for Enhancing High Energy Density in Asymmetric Supercapacitors
Source: Langmuir. 2025 Apr 21;41(16):10469–80. doi: 10.1021/acs.langmuir.5c00378 (PMC12044688; doi:10.1021/acs.langmuir.5c00378)
Supplement: Supplementary file 1 — la5c00378_si_001.pdf [file la5c00378_si_001.pdf]

Supporting Information

Structural Engineering of Core-Shell $Ni_3B@Ni(BO_2)_2$ on V_2MoO_8 (0D@2D/1D) Composites: Advanced Strategies for Enhancing High Energy Density in Asymmetric Supercapacitors

Ahamed Milton[a,1], Abdullah Al Mahmud[a,1], Ramaraj Sukanya[b,1], Raj Karthik[b,1], Eswaran Kamaraj[c,1], Carmel B Breslin[b,*], P. Muhammed Shafi[d,*], Jae-Jin Shim[a,*]

[a]School of Chemical Engineering, Yeungnam University, Gyeongsan, Gyeongbuk 38541, The Republic of Korea.

[b]Department of Chemistry, Maynooth University, Maynooth, Co. KildareW23F2H6, Ireland.

[c]Department of Chemistry, Yeungnam University, Gyeongsan, Gyeongbuk 38541, The Republic of Korea.

[d]Department of Physics, National Institute of Technology Calicut, Calicut, Kerala 673601, India.

Corresponding Authors:
Prof. Jae-Jin Shim (J.-J. Shim)
E-mail: jjshim@yu.ac.kr
Dr. Muhammed Shafi
E-mail: shafiparasseri@gmail.com
Prof. Carmel B Breslin
E-mail: Carmel.Breslin@mu.ie

Number of pages: 14

Number of figures: 4

Number of schemes: 0

Number of tables: 5

Table of Contents

1. Supplementary Experimental Procedures

1.1 Chemicals…………………………………………………………………………. S3

1.2 Graphene Oxide (GO) Synthesis……………………………………………………S3

1.3 Reduced graphene oxide (rGO) synthesis…………………………………………… ….S3

1.4 Physicochemical Characterization Techniques……………………………………… ….S3

1.5 Electrochemical Measurements……………………………………………………… …S4

2. Table S1: Capacitance comparison of the synthesized materias at 1 A g^{-1}………………..S6

3. Table S2: Specific capacitance of synthesized materials at various current densities…….S6

4. Figure S1. Detail CV and GCD curves of (a, b) VMO, (c, d) NB………………………...S7

5. Figure S2. FE-SEM images of NB@NBO/VMO-50 electrode…………………………...S8

6. Figure S3. (a-e) CV curves of at different scan rates showing……………………………S9

7. Table S3: Capacitance of NB@NBO/VMO-50/CC||rGO...S10

8. Table S4: The results of energy and power density for the………………………………S11

9. Figure S4. Ragone plot for asymmetric NB@NBO/VMO-50/CC||rGO..............................S11

10 Table S5: Comparison of the performance of the NB@NBO/VMO//rGO.....................S12

11. References……………………………………………………………………………S13

1. Supplementary Experimental Procedures

1.1 Chemicals

Sulfuric acid (H_2SO_4, 95.0%), hydrogen peroxide (H_2O_2, 30 wt%) and graphite powder (100 mesh) were purchased from Alfa Aesar, Samchun, and Sigma-Aldrich, in that order. Potassium permanganate ($KMnO_4$) and phosphoric acid (H_3PO_4) were purchased from Duksan.

1.2 Graphene Oxide (GO) Synthesis

Graphene oxide (GO) was synthesized using a modified Tour's method. Initially, 360 mL of H_2SO_4 and 40 mL of H_3PO_4 were mixed in a glass beaker under continuous magnetic stirring for 30 minutes to ensure thorough mixing. Subsequently, 3 g of graphite powder was gradually added to the acid mixture. This was followed by the addition of 18 g of $KMnO_4$, and the resulting mixture was magnetically stirred at 50 °C for 12 hr. After the reaction period, the mixture was allowed to cool to room temperature. While maintaining the beaker in an ice bath, 3 mL of H_2O_2 (3%) was added and the mixture was stirred. The solution was then filtered and centrifuged, followed by washing with deionized water until the pH was adjusted to 7. Finally, the obtained material was collected and subjected to freeze-drying for 4 days.

1.3 Reduced Graphene Oxide (rGO) Synthesis

100 mg of GO was dispersed in 60 mL of deionized (DI) water using sonication for 30 minutes. The mixture was then maintained at 180 °C for 6 hours. During this process, the gray-colored GO turned black. The resultant black reduced graphene oxide (rGO) material was collected and dried using a freeze dryer for 4 days.

1.4 Physicochemical Characterization Techniques

The crystal arrangement of the fabricated materials, including NB, VMO rods, and various combinations of NB@NBO/VMO composites, was first analyzed using X-ray diffraction (XRD, PANalytical X'Pert Pro) with a Cu Kα radiation source. The morphology of the composites was observed with a field emission scanning electron microscope (FE-SEM, Hitachi S-4800), accompanied by Energy Dispersive X-ray (EDX) mapping for elemental distribution. Transmission electron microscopy (TEM) and high-resolution TEM (HR-TEM) images were captured using an FEI Tecnai G2 F20 S-TWIN microscope at an accelerating voltage of 200 kV. The oxidation states of the elements present in the NB@NBO/VMO composites were identified using X-ray photoelectron spectroscopy (XPS, Thermo Scientific K-Alpha).

1.5 Electrochemical Measurements

Electrochemical tests, including galvanostatic charge-discharge (GCD), electrochemical impedance spectroscopy (EIS), and cyclic voltammetry (CV), were conducted using an Metrohm Autolab PGSTAT302N in both three-electrode and two-electrode configurations. Cyclic stability was assessed using a multichannel Wonatech WBCS3000L battery cycler. All electrodes were prepared using the slurry coating technique. For electrode fabrication, synthesized NB@NBO/VMO composites, activated carbon black, and PVDF were mixed in a solvent of NMP in a ratio of 85:10:5 to form a slurry. This slurry was applied evenly onto carbon cloth (CC (1 x 1 cm^2)), and electrochemical performance measurements were conducted with these electrodes. The same process was used to prepare electrodes for additional comparison materials. The prepared electrodes were dried in a vacuum oven at 70 °C for 12 hr.

In the two-electrode electrochemical setup, the positive and negative electrodes were separated by commercial Whatman glass fiber filter paper. A 3 M KOH electrolyte solution was used for all electrochemical performance tests. In the three-electrode system, an Ag/AgCl electrode served as the reference electrode, and a Pt plate served as the counter electrode. CV measurements were performed with an operating potential range of 0.0 - 0.5 V at various scan rates, while GCD measurements were conducted from 0.0 - 0.45 V at different current densities. The specific capacitance (C_s) of the as-prepared electrode materials was calculated using the following formula, Eq S1.:

$$C_s = \frac{i\Delta t}{m\Delta V} \tag{S1}$$

where i is the current (A), Δt is the discharge time (s), m is the mass of the active material (g), and ΔV is the potential window (V).

The mass ratio of the positive and negative materials was adjusted based on the principle of charge conservation to enhance the energy density (*ED*) of asymmetric SCs (ASCs), as described by Eq. (S2):

$$m^+/m^- = C^-\Delta V^- \,/\, C^+\Delta V^+ \tag{S2}$$

where, m^+ and m^- represent the active mass loading (g) of the cathode and anode electrodes, respectively; C^+ and C^- are the specific capacitances (F g^{-1}) of the cathode and anode materials, respectively; and V^+ and V^- denote the operational potentials (V) of the cathode and anode electrodes, respectively.

The C_s of the fabricated ASC were estimated using Eq. (S1), where m represents the total mass loading of both the anode and cathode materials.

Additionally, the ED (Wh kg^{-1}) and power density (PD, W kg^{-1}) of the ASC device were determined using Eqs. (S3) and (S4).

$$ED = \frac{1}{2}\frac{CV^2}{(3.6)} \quad \text{(S3)}$$

$$PD = \frac{3600E_D}{\Delta t} \quad \text{(S4)}$$

where, C represents the specific capacitance (F g^{-1}), V denotes the potential window (V), and (Δt) is the discharge time (s) of the ASC.

Table S1: Capacitance comparison of the synthesized materias at 1 A g^{-1}.

**Electrodes**	**Sp. Capacitance (F g^{-1})**
VMO/CC	51.5
NB/CC	584
NB@NBO/VMO-25/CC	319
NB@NBO/VMO-50/CC	698
NB@NBO/VMO-75/CC	411

Table S2: Specific capacitance of synthesized materials at various current densities.

Electrode material /CC	Specific capacitance, C_s (F g^{-1})						
	**1 A g^{-1}**	**2 A g^{-1}**	**3 A g^{-1}**	**4 A g^{-1}**	**5 A g^{-1}**	**10 A g^{-1}**	**15 A g^{-1}**
VMO	51.5	25.3	15.7	11.5	9.0	4.2	3.4
NB	584	550	524.5	505.4	486	426	373
NB@NBO/VMO-25	319	297	279	263	248	189	113
NB@NBO/VMO-50	698	651	618	591	566	461	381
NB@NBO/VMO-75	411	380	359	342	328	267	203

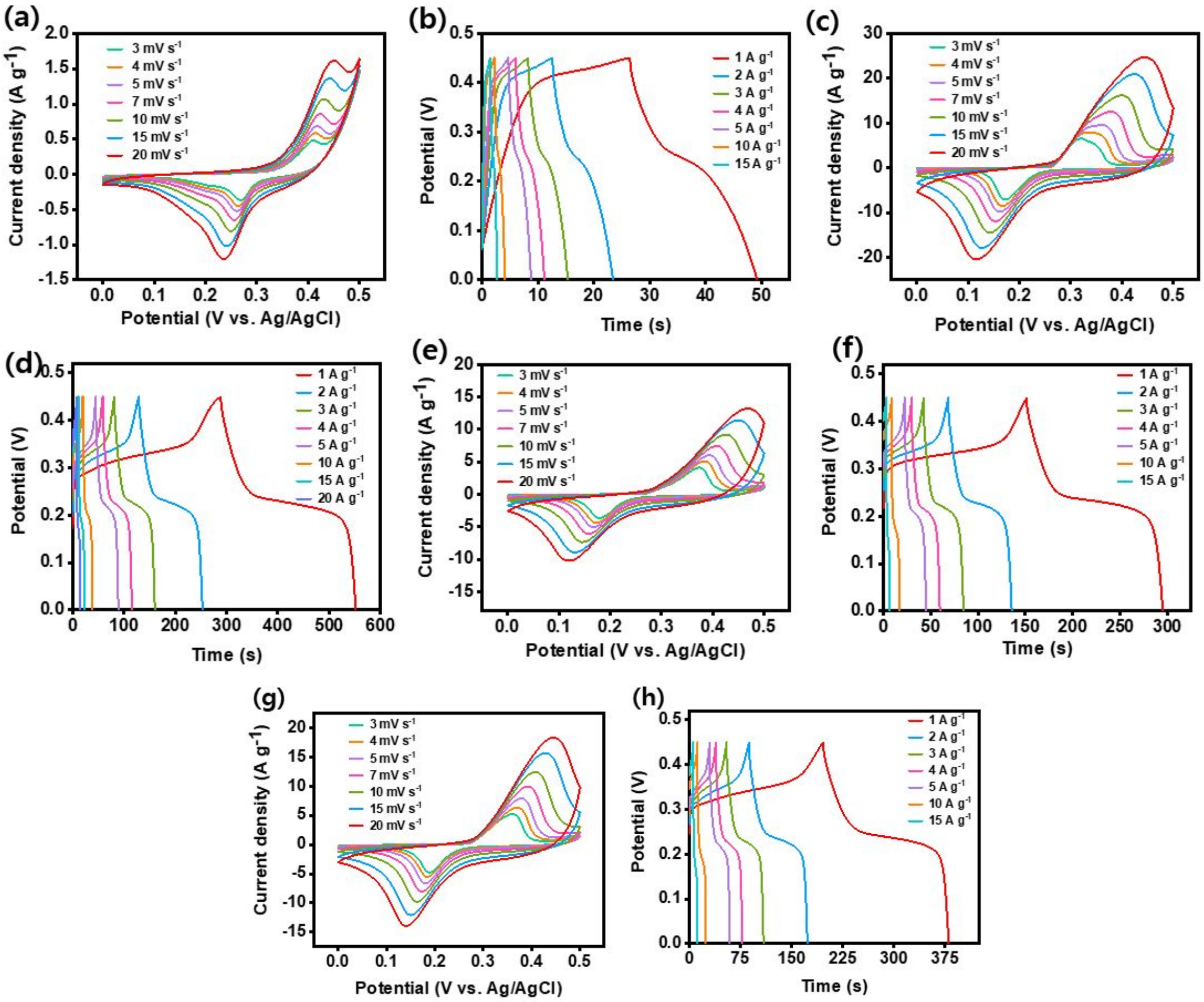

Figure S1. Detail CV and GCD curves of (a, b) VMO, (c, d) NB, (e, f) NB@NBO/VMO-25 and (g, h) NB@NBO/VMO-75.

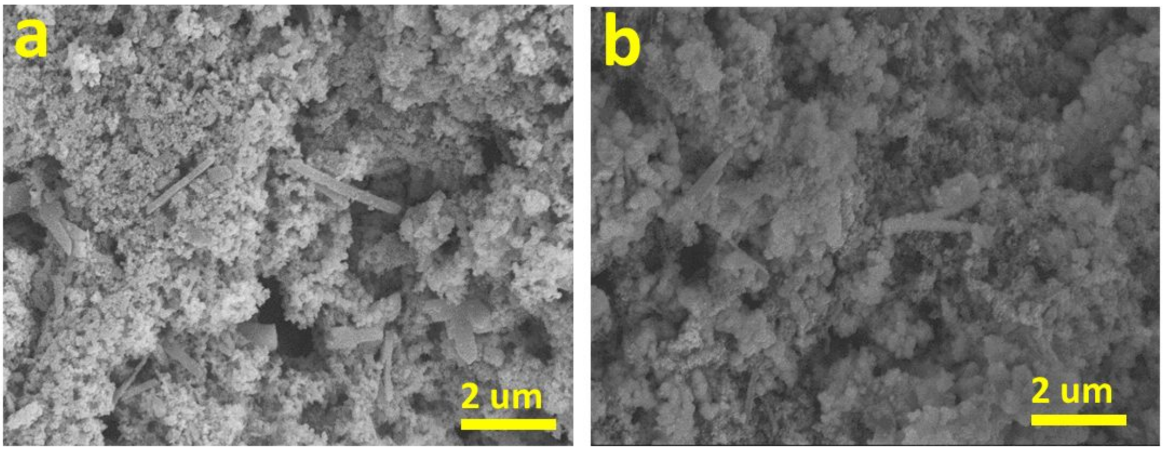

Figure S2. FE-SEM images of NB@NBO/VMO-50 electrode (a) before and (b) after cyclic stability (10000 cycles at 10 A g^{-1}).

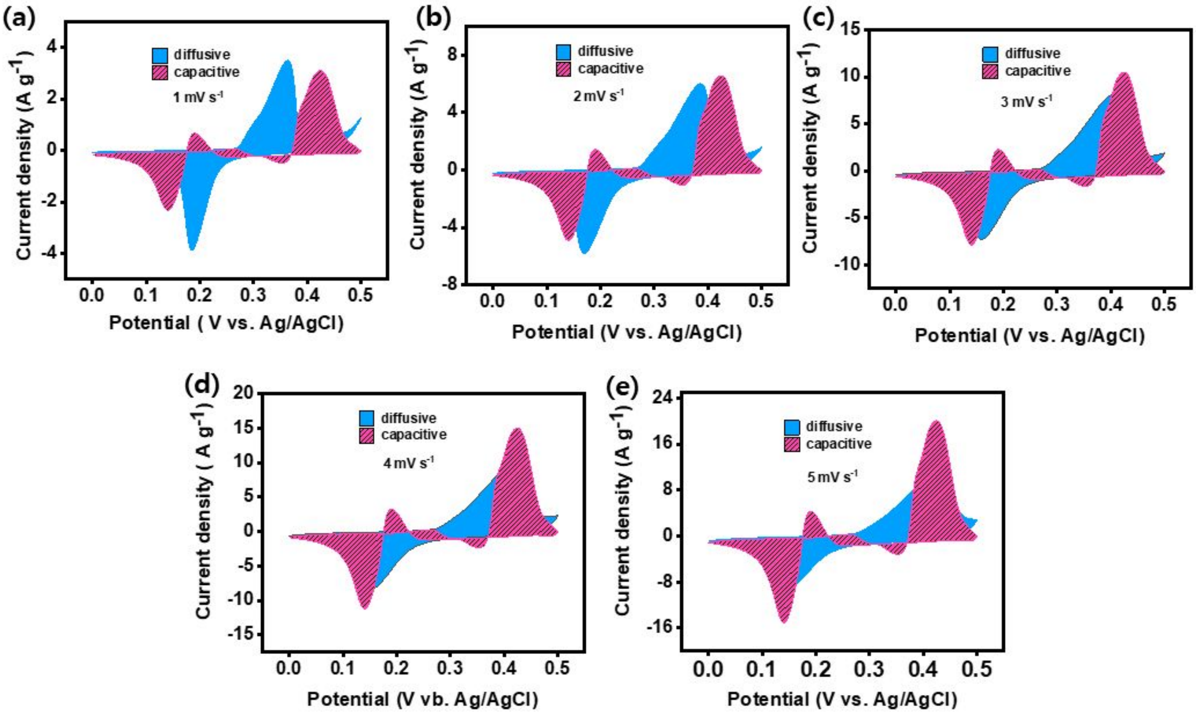

Figure S3. (a-e) CV curves of at different scan rates showing capacitive and diffusive controlled area at NB@NBO/VMO-50/CC.

Table S3: Capacitance of NB@NBO/VMO-50/CC||rGO at different current densities.

**Current density (A g^{-1})**	**Sp. Capacitance (F g^{-1})**
1	114
2	108
3	104
4	101
5	99
10	87
15	76
20	66

Table S4: The results of energy and power density for the NB@NBO/VMO-50/CC||rGO device

Energy Density (Wh kg^{-1})	Power Density (W kg^{-1})
40.5	800
38.4	1600
37	2400
35.9	3200
35.3	4000
30.8	8000
27	12000
23.5	16000

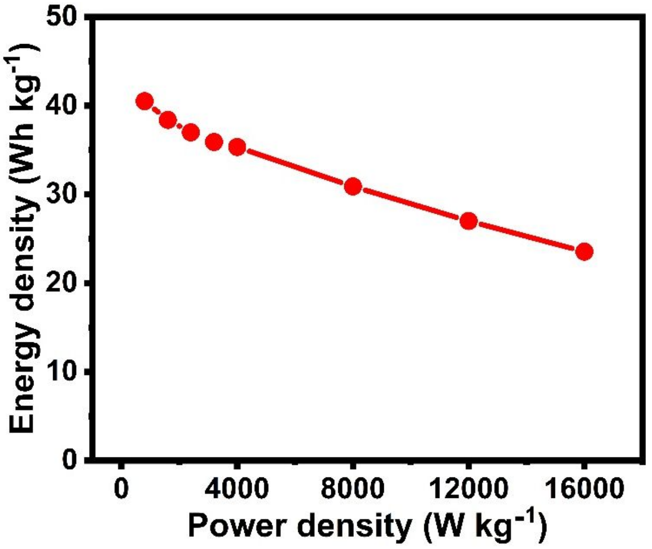

Figure S4. Ragone plot for asymmetric NB@NBO/VMO-50/CC||rGO SC cells.

Table S5: Comparison of the performance of the NB@NBO/VMO//rGO with recently reported SCs.

Electrode Materials	Power Density / W kg^{-1}	Energy Density /Wh kg^{-1}	Ref.
$Ni(OH)_2$@Ni//AC	660	21.8	[S1]
$NiCo_2O_4$//rGO	650	23.9	[S2]
$CoMoO_4$@$NiMoO_4$//AC	267	28.7	[S3]
$Ni_{0.67}Co_{0.33}MoO_4$//RGO	775	25.6	[S4]
$NiCo_2S_4$@$NiCo_2S_4$//rGO	334	24.9	[S5]
CMTs-1000/Ni_2CoS_4//AC	753	28.1	[S6]
CoP/Cu_3P//AC	775.0	28.6	[S7]
$MnCo_2O_4$@NiCo-LDH/NF	160.0	21.3	[S8]
Se-$NiCo_2O_4$/C HNs//AC	415.7	30.1	[S9]
$CoMoO_4$@$NiMoO_4$//AC	267	28.7	[S10]
$Ni_3(BO_3)_2$ NSs@Ti-1.2V//AC	400	30.6	[S11]
$NiMoO_4$/NiCo-LDH//AC	425.5	40.3	[S12]
NCNMS//AC	676.0	30.5	[S13]
Ni_xB/$MnMoO_4$//AC	750	32.5	[37]
NB@NBO/VMO//rGO	800	40.5	This work

References

S1. Su, Y.Z.; Xiao, K.; Li, N.; Liu, Z.Q.; Qiao, S.Z. Amorphous $Ni(OH)_2$@three-dimensional Ni core-shell nanostructures for high capacitance pseudocapacitors and asymmetric supercapacitors. *J. Mater. Chem. A* **2014**, *2*, 13845-13853.

S2. Chen, H.; Jiang, J.; Zhang, L.; Qi, T.; Xia, D.; Wan, H. facilely synthesized porous $NiCo_2O_4$ flowerlike nanostructure for high-rate supercapacitors. *J. Power. Sources* **2014**, 248, 28-36.

S3. Zhang, Z.Q.; Zhang, H.D.; Zhang, X.Y.; Yu, D.Y.; Ji, Y.; Sun, Q.S.; Wang, Y.; Liu, X.Y. Facile synthesis of hierarchical $CoMoO_4$@$NiMoO_4$ core-shell nanosheet arrays on nickel foam as an advanced electrode for asymmetric supercapacitors. *J. Mater. Chem. A* **2016**, *4*, 18578-18584.

S4. Chen, H.C.; Chen, S.; Zhu, Y.Y.; Li, C.; Fan, M.Q.; Chen, D.; Tian, G.L.; Shu, K.Y. Synergistic effect of Ni and Co ions on molybdates for superior electrochemical performance. *Electrochim. Acta* **2016**, *190*, 57-63

S5. Chen, H.C.; Chen, S.; Shao, H.Y.; Li, C.; Fan, M.Q.; Chen, D.; Tian, G.L.; Shu, K.Y. Hierarchical $NiCo_2S_4$ Nanotube@$NiCo_2S_4$ Nanosheet Arrays on Ni Foam for High Performance Supercapacitors. *Chem. Asian. J.,* **2016**, *11*, 248-255.

S6. Wang, K.; Yan, R.; Tian, X.; Wang, Y.; Lei, S.; Li, X.; Yang, T.; Wang, X.; Song, Y.; Liu, Y.; Liu, Z.; Guo. Q. Multi-scale biomass-based carbon microtubes decorated with Ni-Co sulphides nanoparticles for supercapacitors with high-rate performance. *Electrochim. Acta* **2019**, *302*, 78-91.

S7. Liu, W.; Gao, H.; Zhang, Z.; Zheng, Y.; Wu, Y.; Fu, X.; Su, J.; Gao, Y. CoP/Cu_3P heterostructured nanoplates for high-rate supercapacitor electrodes. *Chem. Eng. J.* **2022**, *437*, 135352.

S8. Wang, Y.; Wang, Z.; Zheng, X.; Teng, X.; Xu, L.; Yuan, Y.; Liu, X.; Fu, A.; Li, Y.; Li, H. Core-sheath heterostructure of $MnCo_2O_4$ nanowires wrapped by NiCo-layered double hydroxide as cathode material for high-performance quasi-solid-state asymmetric supercapacitors. *J. Alloys Compd* **2022**, *904*, 164047.

S9. Wang, C.; Sui, G.; Guo, D.; Li, J.; Guo, W.; Chai, D.F. A facile selenic acid etching strategy for designing selenium doped $NiCo_2O_4$/C nanoprisms with hollow/porous structure for advanced asymmetrical supercapacitor. *J. Energy Storage* **2022**, *50*, 104714.

S10. Zhang, Z.; Zhang, H.; Zhang, X.; Yu, D.; Ji, Y.; Sun, Q.; Wang Y.; Liu, X. Facile synthesis of hierarchical $CoMoO_4$@$NiMoO_4$ core-shell nanosheet arrays on nickel foam as

an advanced electrode for asymmetric supercapacitors. *J. Mater. Chem. A* **2016**, *4*, 18578-18584.

S11. Sun, X.; Zhao, K.; Liu, Z.; Feng, Z.; Wang, Z.; Cui, L.; Liu, J. Facile electrodeposition of $Ni_3(BO_3)_2$ nanospheres on Ti mesh for high-performance asymmetric supercapacitors. *J. Energy Storage* **2022**, *55*, 105763.

S12. Cui, S.; Hu, Q.; Sun, K.; Wang, X.; Wang, F.; Hamouda, H.A.; Peng, H.; Ma, G. Nickel-Cobalt-Layered Double Hydroxide Nanosheets Supported on $NiMoO_4$ Nanorods with Enhanced Stability for Asymmetric Supercapacitors. *ACS Appl. Nano Mater.* **2022**, *5*, 6181-6191

S13. Meng, Y.; Liu, J.; Yu, D.; Guo, C.; Liu, L.J.; Hu, Y.; Wang, C.; Zhao, X.; Liu, X. Design of $NiCo_2O_4$@$NiMoO_4$ core-shell nanoarrays on nickel foam to explore the application in both energy storage and electrocatalysis. *Mater. Chem. Front.* **2022**, *6*, 1056-1067.
